# Association of Cytokine Gene Polymorphisms and Their Impact on Active and Latent Tuberculosis in Brazil’s Amazon Region

**DOI:** 10.3390/biom13101541

**Published:** 2023-10-18

**Authors:** Ednelza da Silva Graça Amoras, Thais Gouvea de Morais, Rafaella do Nascimento Ferreira, Samara Tatielle Monteiro Gomes, Francisca Dayse Martins de Sousa, Iury de Paula Souza, Ricardo Ishak, Antonio Carlos Rosário Vallinoto, Maria Alice Freitas Queiroz

**Affiliations:** Virus Laboratory, Institute of Biological Sciences, Federal University of Pará, Belém 66075-110, Brazil; ednelza@hotmail.com (E.d.S.G.A.); ts8.tsga@gmail.com (T.G.d.M.); rafaellanf1@gmail.com (R.d.N.F.); samara_tatielle@yahoo.com.br (S.T.M.G.); msousa.day@gmail.com (F.D.M.d.S.); psouza.iury@gmail.com (I.d.P.S.); rishak@ufpa.br (R.I.); vallinoto@ufpa.br (A.C.R.V.)

**Keywords:** active tuberculosis, latent tuberculosis infection, *Mycobacterium tuberculosis*, polymorphisms, cytokines

## Abstract

Some genetic variations in cytokine genes can alter their expression and influence the evolution of *Mycobacterium tuberculosis* (Mtb) infection. This study aimed to investigate the association of polymorphisms in cytokine genes and variability in plasma levels of cytokines with the development of tuberculosis (TB) and latent tuberculosis infection (LTBI). Blood samples from 245 patients with TB, 80 with LTBI, and healthy controls (n = 100) were included. Genotyping of the *IFNG* +874A/T, *IL6* -174G/C, *IL4* -590C/T, and *IL10* -1082A/G polymorphisms was performed by real-time PCR, and cytokine levels were determined by flow cytometry. Higher frequencies of genotypes AA (*IFNG* +874A/T), GG (*IL6* -174G/C), TT (*IL4* -590C/T), and GG (*IL10* -1082A/G) were associated with an increased risk of TB compared to that of LTBI (*p* = 0.0027; *p* = 0.0557; *p* = 0.0286; *p* = 0.0361, respectively) and the control (*p* = <0.0001, *p* = 0.0021; *p* = 0.01655; *p* = 0.0132, respectively). In combination, the A allele for *IFNG* +874A/T and the T allele for *IL4* -590C/T were associated with a higher chance of TB (*p* = 0.0080; *OR* = 2.753 and *p* < 0.0001; *OR* = 3.273, respectively). The TB group had lower levels of IFN-γ and higher concentrations of IL-6, IL-4, and IL-10. Cytokine levels were different between the genotypes based on the polymorphisms investigated (*p* < 0.05). The genotype and wild-type allele for *IFNG* +874A/T and the genotype and polymorphic allele for *IL4* -590C/T appear to be more relevant in the context of Mtb infection, which has been associated with the development of TB among individuals infected by the bacillus and with susceptibility to active infection but not with susceptibility to latent infection.

## 1. Introduction

Tuberculosis (TB) is caused by *Mycobacterium tuberculosis* (Mtb), which is acquired through the respiratory tract and can cause a primary infection of alveolar macrophages or establish a latent infection [1]. The World Health Organization (WHO) estimates that approximately 10 million people become ill with TB per year worldwide [2].

The evolution of Mtb infection to TB results from a variety of combined factors, such as the virulence mechanisms of the bacillus, the host’s genetics and nutritional status, and vaccination history [3]. Thus, infected individuals may develop latent tuberculosis infection (LTBI), which is asymptomatic and nontransmissible, or TB, which is characterized by the presence of clinical symptoms resulting from lung infection and spread to multiple organs [4]. The World Health Organization (WHO) defines LTBI as a state of persistent immune response to stimulation by Mtb antigens without evidence of clinical disease [5,6]. Approximately one-quarter of the global population has LTBI [5]. Thus, individuals with LTBI represent a reservoir for new cases of active TB [7]. The probability of progression from latent infection to active clinical tuberculosis is determined by bacterial, host, and environmental factors, including: the initial bacillary load, related to the severity of the disease in an index case and the proximity of contact; suppression of cellular immunity induced by the human immunodeficiency virus (HIV); tumor necrosis factor α (TNF-α) inhibitors; glucocorticoids; and organ transplantation [6].

Mtb infection is regulated by two distinct patterns of T helper CD4^+^ cell responses, which result in the production of pro-inflammatory (T helper 1, Th1) and anti-inflammatory (T helper 2, Th2) mediators [8]. Initially, a protective immune response is mounted by natural killer (NK) cells and antigen-specific Th1 cells with the production of IFN-γ, which stimulates the antimicrobial activity of infected alveolar macrophages while organizing the formation of the protective granuloma [8,9,10]. However, Mtb may modulate Th1 cell activation, inducing the synthesis of T helper 2 (Th2) cytokines, which are associated with progressive disease [8,11]. The marked production of suppressive cytokines, such as TGFβ and IL-4, during Mtb infection inhibits phago-lysosome fusion in macrophages and reduces the expression of T lymphocyte-activating molecules [10]. Although the host immune response has been associated with the development of TB, the identification of specific biomarkers that can characterize LTBI is still lacking, as many mediators related to the latency stage also correlate with the acute phase of Mtb infection [12].

Single-nucleotide polymorphisms (SNPs) in the promoter and coding regions of key genes have been associated with susceptibility and/or resistance to Mtb infection. The wild-type *IFNG* +874A/T polymorphism allele is associated with lower IFN-γ synthesis [13], and its presence may impair macrophage activation, thus affecting Mtb infection control [14]. High levels of interleukin 6 (IL-6) are produced in response to Mtb infection by activated monocytes and macrophages [15]. The wild-type *IL6* -174G/C polymorphism is associated with higher levels of IL-6 and has been described as a risk factor for the development of TB [16]. IL-4 and IL-10 are Th2 cytokines involved in the downregulation of IFN-γ expression and therefore have a deleterious effect in patients with Mtb infection [17,18]. *IL4* -560C/T polymorphisms may influence gene transcription and have been associated with increased IL-4 levels [19]. The SNP in *IL10* -1082A⁄G alters the level of IL-10 and interferes with the Th1/Th2 balance [20].

The influence of polymorphisms on the development of diseases seems to depend on factors such as the type of genetic variation (the affected gene and the position where the genetic alteration occurs), the interaction of the effects of various mutations, and, mainly, the ethnic composition of a population [21,22]. The present study aimed to investigate the association of *IFNG* +874A/T, *IL6* -174G/C, *IL4* -590C/T, and *IL10* -1082A/G polymorphisms and plasma levels of cytokines with susceptibility to Mtb, the presence of LTBI, and the development of TB.

## 2. Materials and Methods

### 2.1. Study Population

The study included a group of patients diagnosed with clinical TB (n = 245) attending the João de Barros Barreto University Hospital (HUJBB) in Belém Pará, Brazil. Individuals co-infected with HIV were excluded from the study. Infection was confirmed by X-ray, sputum smear, specific culture of bronchial lavage fluid, and positive biopsy results for Mtb. Patients were examined before starting treatment. The LTBI group was formed of 80 contact individuals with a positive result in the tuberculin skin test (TST) but who never had TB.

The control group consisted of 100 health professionals from the HUJBB who had never presented with TB. These professionals are subjected to periodic occupational examinations every six months. Those who had a negative TST and presented normal results in the following tests were included in the study: chest X-ray, sputum smear microscopy, complete blood count, laboratory test for venereal diseases (VDRL), routine urine analysis, and plasma glucose assessment. All participants in the groups reported having been vaccinated and showing scars from the BCG vaccine.

### 2.2. Sample Collection and Storage

Blood samples (10 mL) were collected by intravenous puncture using a vacuum collection system containing EDTA as an anticoagulant. Samples were centrifuged to isolate plasma and leukocytes, which were used for genomic DNA extraction to investigate gene polymorphisms. Samples were stored in a freezer at −70 °C until use.

### 2.3. DNA Extraction

Genomic DNA was extracted from peripheral blood leukocytes using a Puregene kit (Puregene, Gentra Systems, Inc., Minneapolis, MN, USA) according to the manufacturer’s protocol, which included the following steps: cell lysis, protein precipitation, DNA precipitation, and hydration. After extraction, the DNA obtained was quantified by spectrophotometry in a NanoDrop® system (Thermo Fisher Scientific, Wilmington, DE, USA) following the manufacturer’s recommendations.

### 2.4. Genotyping

Polymorphism genotyping was performed by real-time PCR using the StepOnePlus™ Real-Time PCR System (Thermo Fisher, Carlsbad, CA, USA). Assay systems containing primers and probes specific for amplification of the sequence of each target were used (Thermo Fisher, Carlsbad, CA, USA). The following polymorphisms with their respective commercial assays were investigated: *IL4* -590C/T (rs2243250; C__16176216_10); *IL6* -174G/C (rs1800795; C_1839697_20); and *IL10* -1082A/G (rs1800896; C_1747360_10). For identification of *IFNG* +874A/T genotypes, primers and probes were used [23]: primers (IFNG-F: 5′-TTC AGA CAT TCA CAA TTG ATT TTA TTC T-3; IFNG-R: 5′−CCC CCA ATG GTA CAG GTT TC-3′); and probes (FAM-AAAATCAAATCTCACACACACA-MGB; VIC-AAAATCAAATCACACACACACA-MGB).

For each reaction, TaqMan® Universal PCR Master Mix [2X], [20X] TaqMan® Assay, and 20 ng of DNA were used in a final reaction volume of 10 µL. The following temperature cycles were used in the amplification reactions: 60 °C for 30 s, followed by 95 °C for 10 min, and 50 cycles of 92 °C for 30 s and 60 °C for 1 min and 30 s.

### 2.5. Plasma Cytokine Measurement

Cytokine levels were quantified by flow cytometry using a Human Th1/Th2/Th17 Cytometric Bead Array (CBA) Kit (BD Biosciences, San Diego, CA, USA) in a BD FACS Canto II instrument. All procedures followed the manufacturer’s instructions; the method is based on beads conjugated to a capture antibody, i.e., six populations of beads with different fluorescence intensities, conjugated to a capture antibody specific for each cytokine, mixed to form the CBA, and read in channel FL-3 in the flow cytometer. The bead populations were recorded on the basis of their respective fluorescence intensities, from the least bright to the brightest (IL-17 < IFN-γ < TNF-α < IL-10 < IL-6 < IL-4 < IL-2).

### 2.6. Tuberculin Skin Test (TST)

Control subjects submitted to a TST, performed by applying an intradermal injection of 0.1 mL (0.04 mcg) of PPD RT-23 (Mantoux, 2 UT/0.1 mL) in the middle third of the anterior surface of the left forearm, at an angle of 5 to 15 degrees, until the formation of a papule. The reading was performed 48 to 72 h after application using a specific millimeter ruler, measuring the largest transverse diameter of the induration perpendicular to the forearm. The results with induration greater than or equal to 5 mm were considered to indicate a positive PPD test [24].

### 2.7. Statistical Analysis

The data obtained was entered into a database in Microsoft Office Excel 2020 (Microsoft, Redmond, WA, USA). The genotypic and allelic frequencies were compared between the groups using the chi-square and G tests. The odds ratio (OR) was used to evaluate the homozygous genotypes for each polymorphism (wild-type and polymorphic, or vice versa) that represented a risk of developing the different clinical forms of TB. Variations in the plasma levels of the cytokines between the groups were evaluated using the nonparametric Kruskal-Wallis test and Dunn´s post-test. Violin charts were used to show the dispersion of cytokine levels between the groups investigated. All tests were performed using BioEstat version 5.3 and GraphPad Prism 8.0 (GraphPad Software, San Diego, CA, USA), and *p* values < 0.05 were considered to indicate significance.

## 3. Results

The characteristics of the studied groups showed that among patients with TB, 59.0% were male, 82.0% had the pulmonary form, and 18.0% had the extrapulmonary form of the disease, with a mean age of 41.2 years. In patients with LTBI, 57.5% were male, and the mean age was 38.4 years. In healthy controls, 58.0% were male, and the mean age was 39.7 years (Table 1).

The comparison of the allelic and genotypic frequencies of the *IFNG* +874A/T polymorphism between the TB and LTBI groups showed that the wild-type AA genotype (*p* = 0.0027; OR = 3.6104, 95% CI 1.36–9.60) and the A allele (*p* < 0.0001; OR = 2.1649, 95% CI 1.48–3.1) were more frequent in the TB group. In the comparison between the TB and control groups, an association of the wild-type AA genotype (*p* < 0.0001; OR: 5.5156, 95% CI 2.45–11.9) and the A allele (*p* < 0.0001; OR = 2.2790, 95% CI 1.60–3.22) with the TB group was observed. Between the LTBI and HC groups, there was no significant difference between genotypic and allele frequencies (Table 2).

The comparison of *IL6* -174G/C genotypes between the evaluated groups showed that there was no significant difference in genotypic and allelic frequencies between the TB and LTBI groups (*p* > 0.05), but when compared to the control group, the TB group had a higher frequency of the GG genotype (*p* = 0.0021; OR = 4.3955, 95% CI 1.46–13.22) and G allele (*p* = 0.0003; OR = 2.1128, 95% CI 1.41–3.14). There was no significant difference in genotype and allele frequencies between the LTBI and HC groups (Table 2).

For the *IL4* -590C/T polymorphism, a higher frequency of the TT genotype (*p* = 0.0286; OR = 2.800, 95% CI 1.26–6.22) and of the T allele (*p* = 0.0029; OR = 1.6300, 95% CI 1.12–2.35) was observed in the TB group than in the LTBI group. The comparison of the TB and control groups showed that the TT genotype and the T allele were significantly more frequent in the TB group (*p* = 0.01655; OR = 2.7097, 95% CI 1.31–5.60 and *p* = 0.0013; OR = 1.6213, CI 1.58–2.27, respectively). Between the LTBI and HC groups, differences between genotype and allele frequencies were not significant (Table 2).

Genotyping of the *IL10* +1082G/A polymorphism showed that the GG genotype was significantly more frequent in the TB group than in the LTBI (*p* = 0.0132) and control groups (*p* = 0.0361). The allele frequencies did not show significant differences among the evaluated groups. Genotypic and allele frequencies did not show significant differences between the LTBI and HC groups (Table 2).

We evaluated whether allelic combinations for the *IFNG* +874A/T, *IL6* -174G/C, *IL4* -560C/T, and *IL10* +1082A/G polymorphisms contribute to risk or protection from active Mtb infection. Of the 16 possible combinations, there was an association between the A for *IFNG* +874A/T and T for *IL4* -560C/T alleles with TB compared to LTBI, especially in the combinations AGTA (*p* = 0.0008; OR  =  2.753; 95% CI 1.52–4.96). The A allele for *IFNG* +874A/T and T for *IL4* -560C/T were associated with the risk of TB when compared to the control, mainly in the AGTA (*p* < 0.0001; OR = 3.273; 95% CI 1.85–5) (Table 3).

In contrast, some combinations of the T polymorphic allele for the *IFNG* +874A/T variation appeared to protect against TB when compared to the LTBI group, especially the combinations TGCA (*p* = 0.0103; OR = 0.396; 95% CI 0.20–0.77), TGCG (*p* = 0.0352; OR = 0.344; 95% CI 0.14–0.86), TGTG (OR = 0.357; 95% CI 0.16–0.78), and TCTA (*p* = 0.0157; OR = 0.266; 95% CI 0.09–0.80). Similarly, some combinations with the T allele for *IFNG* +874A/T appear associated with a lower risk of TB in comparison to healthy controls, including TCCA (*p* = 0.0200; OR = 0.245; 95% CI 0.08–0.75), TCTA (*p* = 0.0062; OR = 0.233; 95% CI 0.08–0.65), and TCTG (*p* = 0.0348; OR = 0.388; 95% CI 0.17–0.88; Table 3).

The comparison of the LTBI group and the control group showed no significant difference for any of the combinations evaluated (*p* > 0.05).

**Figure 1:**
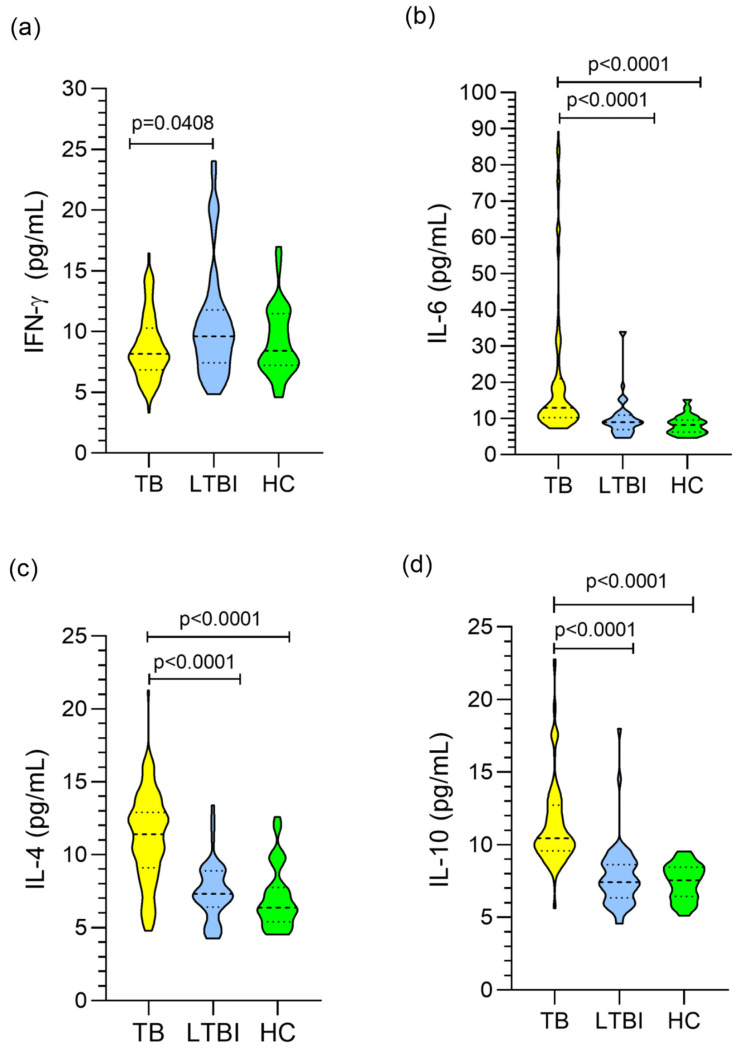
Comparison of the levels of the cytokines IFN-γ (**a**), IL-6 (**b**), IL-4 (**c**), and IL-10 (**d**) among the TB, LTBI, and healthy control (HC) groups. Kruskal-Wallis test and Dunn’s post-test.

Assessment of differences in cytokine levels between groups showed that the LTBI group had higher levels of IFN-γ similar to the control group (*p* = 0.0408; Figure 1a). The levels of the cytokines IL-6, IL-4, and IL-10 were higher in the TB group than in the LTBI and control groups (*p* < 0.0001; Figure 1b–d).

**Figure 2:**
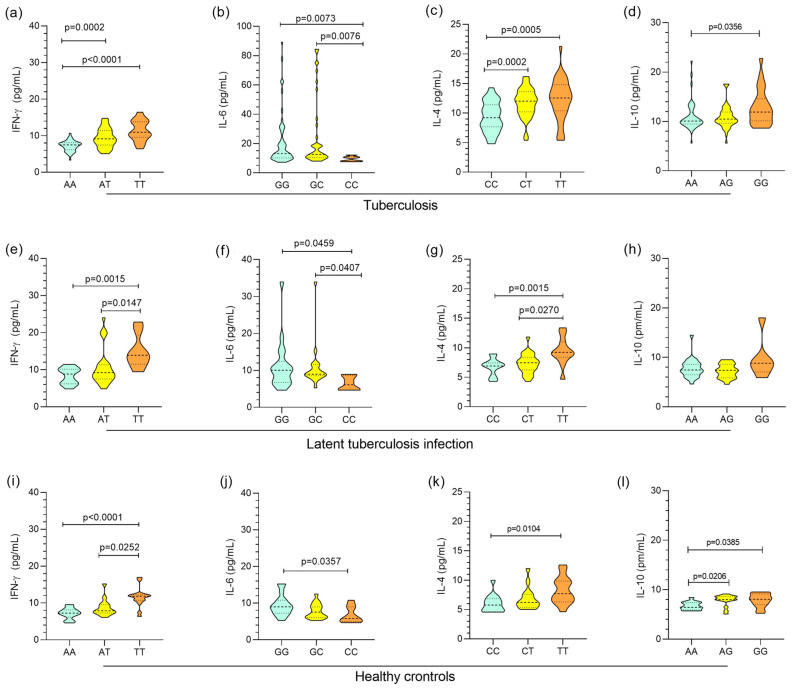
Comparison of the levels of the cytokines IFN-γ, IL-6, IL-4, and IL-10 among the genotypes of the polymorphisms *IFNG* +874A/T, *IL6* -174G/C, *IL4* -560C/T, and *IL10* +1082A/G in the TB (**a**–**d**), LTBI (**e**–**h**), and healthy control (**i**–**l**) groups. Kruskal-Wallis test and Dunn´s post-test.

In Figure 2, the plasma levels of the cytokines IFN-γ, IL-6, IL-4, and IL-10 were grouped by genotype, and the levels showed a similar profile in the investigated groups. The AA, AT, and TT genotypes for *IFNG* +874A/T were associated with low, intermediate, and high levels of IFN-γ, respectively, in the active TB (Figure 2a), LTBI (Figure 2e), and control (Figure 2i) groups. IL-6 concentrations were significantly higher in carriers of the GG genotype for *IL6* -174G/C than in individuals with the CC genotype in the active TB (Figure 2b), LTBI (Figure 2f), and control (Figure 2j) groups. A comparison of IL-4 levels showed that individuals with the TT polymorphic genotype for *IL4* -560C/T had higher levels than carriers of the wild-type CC genotype in the active TB (Figure 2c), LTBI (Figure 2g), and control (Figure 2k) groups. Higher concentrations of *IL-10* were observed in individuals with the polymorphic genotype GG for *IL10* +1082A/G than in those with the wild-type AA genotype in the active TB (Figure 2d), LTBI (Figure 2h), and control (Figure 2l) groups.

**Figure 3:**
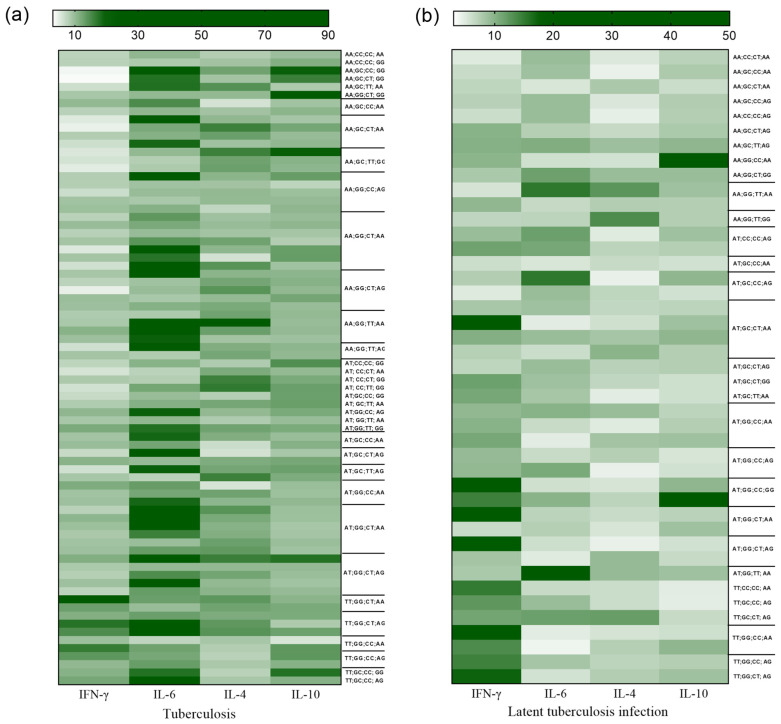
Analysis of the concentrations of the cytokines IFN-γ, IL-6, IL-4, and IL-10 according to the genotypes of the polymorphisms *IFNG* +874A/T, *IL6* -174G/C, *IL4* -560C/T, and *IL10* -1082A/G in groups with TB (**a**) and LTBI (**b**).

As shown in the heatmap, there is an inversion of Th1 and Th2 cytokine concentrations between the groups with active TB (Figure 3a) and LTBI (Figure 3b). However, the cytokine concentrations were related to certain genotypes in both evaluated groups. In the subsets plotted for active TB, an association of lower concentrations of IFN-γ with higher levels of the cytokines IL-6, IL-4, and IL-10 was observed (Figure 3a). Conversely, in the LTBI group, there was an association between higher levels of IFN-γ and lower concentrations of IL-6 and Th2 cytokines (Figure 3b).

## 4. Discussion

Variations in the levels of specific cytokines determine the susceptibility and progression of Mtb infection or containment of the infection. Some cytokines resulting from the activation of T lymphocytes and phagocytic cells, such as IFN-γ and TNF-α, are efficient in controlling infection, while others may contribute to the survival and growth of Mtb or mediate susceptibility to tuberculosis, including IL-4, TGF-β, and IL-10 [25]. As genes encoding cytokines such as *IFNG*, *IL4*, *IL6*, and *IL10* are highly polymorphic, some genetic variations may influence the expression of cytokines and contribute to regulating resistance or susceptibility to TB [20,26].

The study showed an association between the frequencies of the wild genotype for *IFNG* +874A/T and *IL6* -174G/C and the polymorphic genotype of *IL4* -560C/T and *IL10* +1082A/G with TB. IFN-γ levels were more reduced in individuals with TB and higher in the LTBI group, while IL-6, IL-4, and IL-10 levels were higher in individuals with TB and reduced in the LTBI group. There was no statistical difference in the frequencies of investigated polymorphisms and cytokine levels between the LTBI and control groups. IFN-γ levels were more reduced in individuals with the AA genotype for *IFNG* +874A/T, and the levels of IL-6, IL-4, and IL-10 were higher in carriers of the GG, TT, and GG genotypes for the variations *IL6* -174G/C, *IL4* -560C/T, and *IL10* +1082A/G, respectively.

Although this study shows interesting results related to the isolated evaluation of each of the polymorphisms and their association and the influence of polymorphisms on cytokine levels in different groups, the small sample size of the LTBI and control groups represents a limiting factor in the study. The characterization of the LTBI group was also a limitation of the study, since this LTBI group was selected due to the positive result of the TST test associated with the absence of TB symptoms. The interferon-gamma release assay (IGRA) would be a more appropriate test to define the LTBI group, as it uses Mtb-specific antigens that induce the production of IFN-γ in individuals with previous contact with Mtb, and the result is not influenced by BCG vaccination or exposure to most non-tuberculous mycobacteria (NMT) infections [27,28]. However, according to the WHO, both the TST and IGRA can be used as screening tests for LTBI in countries with high TB endemicity, such as Brazil [29]. According to the Manual of Recommendations for Tuberculosis Control in Brazil (Ministry of Health), the diagnosis of LTBI can be made by TST or IGRA; however, IGRA is recommended only for specific groups, such as HIV-infected individuals and children, not being routinely used to identify LTBI in the general population, as it is less expensive, widely available, and simpler to perform for health professionals [30,31]. As the population included in the study does not meet the criteria for the use of IGRA, the diagnosis was made by TST.

The analysis of the *IFNG* +874A/T polymorphism between the TB and LTBI groups suggested that the increased frequency of the wild-type AA genotype represented a 3-fold greater chance of the individual developing TB among those infected with Mtb; between the TB and HC groups, the presence of the AA genotype was associated with a 5-fold greater chance of the individual acquiring infection and developing the disease.

The wild-type AA genotype is associated with lower cytokine levels and may contribute to the establishment of TB. Other studies have observed an association of the wild-type genotype and allele with TB in different populations [32,33,34]. IFN-γ is fundamental to the protective immune response against Mtb, which is mediated by CD4^+^ Th1 cells [35]. This cytokine has a major impact on the physiology of infected macrophages and on the biology of phagosomes [36]. Elevated cytokine levels may also promote greater FASL activation and enhance the apoptosis of infected cells [37]. However, a deficient IFN-γ response is associated with susceptibility to and severity of TB, as it reduces the activation of T-cell responses and the detection of infected cells [14,38].

The presence of the wild-type GG genotype and the G allele for the *IL6* -174G/C polymorphism was associated with a 4-fold increased risk for infection and development of TB when comparing individuals with TB and the healthy control. The GG and CC genotypes were associated with higher and lower levels of cytokines, respectively. Our results corroborate a previous study in which the presence of the polymorphic genotype CC was associated with a lower risk of TB [39,40]. However, in a study performed in India, the CC genotype was associated with TB [41], in contrast to our results. It is possible that this divergence is related to the ethnic differences in the population. IL-6 has been identified as a key driver of TB severity and is associated with lung inflammation and lung damage. The cytokine, which is produced by macrophages, inhibits IFN-γ signaling, contributing to the severe disease phenotype [42,43].

The TT polymorphic genotype for *IL4* -560C/T was associated with a greater chance (almost three times) of the individual developing TB among those infected by Mtb (TB vs. LTBI) and of having the infection and developing disease (comparison between the TB and control groups). As the TT genotype was related to higher levels of the cytokine IL-4, this result corroborates information previously described [19,44]. Because the polymorphism induces an increase in IL-4 production, it favors a Th2 immune response and antibody production, which is not an efficient response to control Mtb infection and inhibits the differentiation of Th1 cells, which are effective in combating the bacterium. The *IL4* -590C/T polymorphism might affect the ability of macrophages to inhibit infection caused by the bacillus [17]. In addition, increased IL-4 (i) impaired antimicrobial activity due to reduced TNF-α-mediated apoptosis of infected cells and decreased activity of iNOS (and therefore decreased levels of nitrogen and oxygen intermediates), (ii) increased iron availability for intracellular Mtb, and (iii) increased proliferation of FOXP3+ regulatory T cells, favoring the immunopathogenesis of TB [45].

The GG polymorphic genotype of *IL10* -1082A/G was associated with higher cytokine levels in all studied groups, and the highest frequency was observed in patients with TB. Other studies have observed this same association with TB in different populations [20,46]. IL-10 has been reported to limit the effector immune response to Mtb infection through immunosuppressive mechanisms [47]. Production of this cytokine by human macrophages infected with Mtb inhibits phagosome maturation, resulting in impaired bacterial clearance [48] and increased chances of developing active TB [49,50].

Considering that the SNPs are associated with changes in cytokine levels, our results suggest that these polymorphisms may interact synergistically, decreasing the signaling of the Th1 response and therefore interfering with effector mechanisms, leading to a significant enhancement of the pathogenesis of TB [51,52]. In this context, the combination of the allele related to low levels of IFN-γ (A) and high levels of IL-4 (T) was predominantly associated with the risk of being infected and developing TB (when comparing TB with the HC condition), especially in the AGTA (OR = 3.273) and ACTG (OR = 5.038) combinations. These combinations (AGTA; OR = 2.753) and (ACTG; OR = 4.000) were also associated with the risk of developing TB among those infected with Mtb (in the comparison between TB and LTBI groups). These results suggest that genetic variations may contribute to promoting changes in the levels of Th1 and Th2 cytokines. 

An inversion of Th1 and Th2 cytokine concentrations was observed between the TB and LTBI groups. In TB, lower levels of IFN-γ and higher concentrations of IL-6, IL-4, and IL-10 were observed. Conversely, the LBTI condition was associated with higher levels of IFN-γ and lower concentrations of IL-6 and Th2 cytokines. Previous studies have shown that Mtb modulates the effector mechanisms of the immune response, such as decreasing IFN-γ production, impairing the activation of alveolar macrophages, and favoring the differentiation of Th2 cells with an increase in the levels of the primary cytokines IL-4 and IL-10 [52]. Furthermore, Mtb also increases IL-6 production in infected macrophages, reducing IFN-γ effector signaling and contributing to an impaired cellular immune response and impaired infection eradication [53,54].

LTBI appears to result in a balance between the host’s immune response and Mtb activity. Most infected people present a robust Th1 immune response, culminating in the formation of a granulomatous lung lesion that contains the infection and prevents the occurrence of active disease but is not sufficient to eliminate the bacillus [55]. This study showed that individuals with LTBI had a higher protective immune response against Mtb infection than those who had active disease, which was mainly influenced by the *IFNG* +874A/T and *IL4* -590C/T polymorphisms. Furthermore, no differences were observed in the frequencies of polymorphisms and cytokine levels between the LTBI and control groups, suggesting that the genetic variations evaluated are not associated with susceptibility to LTBI. Thus, genetic variations in these cytokine genes may promote susceptibility to active infection and the establishment of TB symptom manifestations. These results reinforce that the immune responses generated against Mtb need to be adequately balanced to eliminate or promote the latency state of the bacillus [51,56,57]. Approximately 5 to 10% of individuals with LTBI progress to active infection during their lifetime [5]. The search for prognostic biomarkers that can predict the risk of active TB in individuals with LTBI is of enormous value for TB control. The identification of a transcriptional profile and gene expression signature that distinguished individuals with active TB from those with LTBI highlighted the expression of *IFNG* as a possible biomarker of differentiation between active and latent forms of Mtb infection [58].

## 5. Conclusions

The genetic variations investigated were shown to influence the levels of the cytokines IFN-γ, IL-6, IL-4, and IL-10; however, the genotype and wild-type allele for *IFNG* +874A/T and the genotype and polymorphic allele for *IL4* -590C/T appear to be more relevant in the context of Mtb infection, which has been associated with the development of TB among individuals infected by the bacillus and with susceptibility to active infection but not with susceptibility to latent infection.

## Figures and Tables

**Table 1:** Characteristics of the study populations.

	TB n = 245	LTBI n = 80	HC n = 100
Age, mean (SD)	41.2 (10.1)	38.4 (9.4)	35.7 (11.6)
Sex, n (%) Female Male	100 (41.0) 145 (59.0)	34 (42.5) 46 (57.5)	42 (42.0) 58 (58.0)
Location, n (%) TB, pulmonary TB, extrapulmonary	201 (82.0) 44 (18.0)	-	-

TB: patients with active tuberculosis; LTBI: subjects with latent tuberculosis infection; HC: healthy controls.

**Table 2:** Distribution of allelic and genotypic frequencies in the TB, LTBI, and healthy control groups.

Alleles and Genotypes	TB	LTBI	HC	*p*	OR
	n = 245	n = 80	n = 100		(95% IC)
	n (%)	n (%)	n (%)		
*IFNG* +874A/T					
AA	139 (56.7) ^†^	22 (27.5)	33 (33.0)	0.0027 ^a∆^	3.6104 (1.36–9.60) ^a^
					*p* = 0.0168
AT	92 (37.55)	50 (62.5) ^†^	49 (49.0)	<0.0001 ^b∆^	5.5156 (2.45–11.9) ^b^
					*p* < 0.0001
TT	14 (5.71)	8 (10.0)	18 (18.0) ^†^	0.1436 ^c∆^	-
	
* A	0.755	0.588	0.571	<0.0001 ^a∆^	2.1649 (1.48–3.15) ^a^
					*p* < 0.0001
* T	0.245	0.412	0.429	<0.0001 ^b∆^	2.2790 (1.60–3.22) ^b^
					*p* < 0.0001
				0.8112 ^c∆^	-
*IL6* -174G/C					
GG	178 (72.7)	53 (66.3)	54 (54.0)	0.0557 ^a∞^	-
GC	61 (24.9)	21 (26.2)	38 (38.0)	0.0021 ^b∞^	4.3955 (1.46–13.22) ^b^
					*p* = 0.0118
CC	6 (2.4)	6 (7.6)	8 (8.0)	0.2222 ^c∞^	-
	
* G	0.851	0.794	0.73	0.9374	-
* C	0.149	0.206	0.27	0.0003 ^b∆^	2.1128 (1.41–3.14) ^b^
					*p* < 0.0001
				0.1603 ^c∆^	-
*IL4* -560C/T					
CC	62 (25.3)	31 (38.8) ^†^	39 (39.0) ^†^	0.0286 ^a∆^	2.8000 (1.26–6.22) ^a^
					*p* = 0.0165
CT	127 (51.8)	39 (48.7)	48 (48.0)	0.01655 ^b∆^	2.7097 (1.31–5.60) ^b^
					*p* = 0.0099
TT	56 (22.9) ^†^	10 (12.5)	13 (13.0)	0.9927 ^c∆^	-
	
* C	0.512	0.631	0.63	0.0029 ^a∆^	1.6300 (1.12–2.35) ^a^
					*p* = 0.0114
* T	0.488	0.369	0.37	0.0013 ^b∆^	1.6213 (1.58–2.27) ^b^
					*p* = 0.0062
				0.9805 ^c∆^	-
*IL10* -1082A/G					
AA	148 (60.4) ^†^	38 (47.5)	50 (50.0)	0.0361 ^a∆^	-
AG	72 (29.4)	36 (45.0) ^†^	45 (45.0) ^†^	0.0132 ^b∆^	-
GG	25 (10.2)	6 (7.5)	5 (5.0)	0.7758 ^c∆^	-
* A	0.746	0.703	0.738	0.3368 ^a∆^	-
* G	0.254	0.297	0.262	0.8903 ^b∆^	-
				0.6020 ^c∆^	-

TB: patients with active tuberculosis; LTBI: subjects with latent tuberculosis infection; HC: healthy controls. n: number of individuals; * alleles; ^a^ TB vs. LTBI; ^b^ TB vs. HC; ^c^ LTBI vs. HC; ^∆^ chi-squared test; ^∞^ G test; ^†^ residue analysis; OR: odds ratio.

**Table 3:** Association between allelic combinations of polymorphisms *IFNG* +874A/T, *IL6* -174G/C, *IL4* -560C/T, and *IL10* -1082A/G in the TB, LTBI, and healthy control groups.

	*IFNG* +874	*IL6* -174	*IL4* -560	*IL10* +1082	TB	LTBI	HC	TB vs. LTBI OR (95% CI)	TB vs. HC OR (95% CI)
1	A	G	C	A	163	53	64	0.998 (0.67–1.44) *p* = 0.9965	1.042 (0.73–1.48) *p* = 0.8900
2	A	G	C	G	27	6	7	1.473 (0.59–3.63) *p* = 0.5256	1.588 (0.68–3.70) *p* = 0.3764
3	A	G	T	A	104	14	15	2.753 (1.52–4.96) * p = 0.0008	3.273 (1.85–5.78) * p < 0.0001
4	A	G	T	G	35	8	10	1.348 (0.61–2.98) *p* = 0.5506	1.356 (0.65–2.81) *p* = 0.5166
5	A	C	C	A	14	6	7	0.742 (0.28–1.96) *p* = 0.7361	0.817 (0.31–2.01) *p* = 0.8213
6	A	C	C	G	7	2	3	1.126 (0.23–5.47) *p* = 0.8064	0.941 (0.24–3.67) *p* = 0.7916
7	A	C	T	A	14	2	8	2.285 (0.51–10.10) *p* = 0.4120	0.698 (0.28–1.69) *p* = 0.5742
8	A	C	T	G	24	2	2	4.00 (0.93–17.11) *p* = 0.0743	5.038 (1.17–21.52) * p = 0.0280
9	T	G	C	A	21	16	12	0.396 (0.20–0.77) * p = 0.0103	0.693 (0.33–1.43) *p* = 0.4282
10	T	G	C	G	10	9	7	0.344 (0.14–0.86) * p = 0.0352	0.568 (0.21–1.51) *p* = 0.3820
11	T	G	T	A	36	9	17	1.307 (0.62–2.77) *p* = 0.6036	0.843 (0.46–1.54) *p* = 0.6909
12	T	G	T	G	14	12	11	0.357 (0.16–0.78) * p = 0.0157	0.499 (0.22–1.12) *p* = 0.1362
13	T	C	C	A	5	4	8	0.396 (0.11–1.49) *p* = 0.3048	0.245 (0.08–0.75) * p = 0.0200
14	T	C	C	G	6	5	8	0.378 (0.11–1.26) *p* = 0.1958	0.294 (0.10–0.85) * p = 0.0382
15	T	C	T	A	6	7	10	0.266 (0.09–0.80) * p = 0.0292	0.233 (0.08–0.65) * p = 0.0062
16	T	C	T	G	12	5	12	0.815 (0.28–2.34) *p* = 0.9220	0.388 (0.17–0.88) * p = 0.0348

TB: patients with active tuberculosis; LTBI: subjects with latent tuberculosis infection; HC: healthy controls. OR: Odds ratio. * *p* < 0.05.

## Data Availability

The data analyzed in this study are included within the paper.
